# ROS Regulation and Antioxidant Responses in Plants Under Air Pollution: Molecular Signaling, Metabolic Adaptation, and Biotechnological Solutions

**DOI:** 10.3390/antiox14080907

**Published:** 2025-07-24

**Authors:** Muhammad Junaid Rao, Mingzheng Duan, Muhammad Ikram, Bingsong Zheng

**Affiliations:** 1 National Key Laboratory for Development and Utilization of Forest Food Resources, Zhejiang A & F University, Hangzhou 311300, China; bszheng@zafu.edu.cn; 2 College of Agronomy and Life Sciences, Zhaotong University, Zhaotong 657000, China; 3 MOA Key Laboratory of Crop Ecophysiology and Farming System in the Middle Reaches of the Yangtze River, College of Plant Science & Technology, Huazhong Agricultural University, Wuhan 430070, China; ikram1996@webmail.hzau.edu.cn

**Keywords:** air pollution, reactive oxygen species, antioxidants, transcription factors, phytohormones, secondary metabolites, oxidative stress

## Abstract

Air pollution acts as a pervasive oxidative stressor, disrupting global crop production and ecosystem health through the overproduction of reactive oxygen species (ROS). Hazardous pollutants impair critical physiological processes—photosynthesis, respiration, and nutrient uptake—triggering oxidative damage and yield losses. This review synthesizes current knowledge on plant defense mechanisms, emphasizing the integration of enzymatic (SOD, POD, CAT, APX, GPX, GR) and non-enzymatic (polyphenols, glutathione, ascorbate, phytochelatins) antioxidant systems to scavenge ROS and maintain redox homeostasis. We highlight the pivotal roles of transcription factors (MYB, WRKY, NAC) in orchestrating stress-responsive gene networks, alongside MAPK and phytohormone signaling (salicylic acid, jasmonic acid, ethylene), in mitigating oxidative stress. Secondary metabolites (flavonoids, lignin, terpenoids) are examined as biochemical shields against ROS and pollutant toxicity, with evidence from transcriptomic and metabolomic studies revealing their biosynthetic regulation. Furthermore, we explore biotechnological strategies to enhance antioxidant capacity, including overexpression of ROS-scavenging genes (e.g., *TaCAT3*) and engineering of phenolic pathways. By addressing gaps in understanding combined stress responses, this review provides a roadmap for developing resilient crops through antioxidant-focused interventions, ensuring sustainability in polluted environments.

## 1. Introduction

In recent decades, the global trends of industrialization and rapid urbanization have triggered environmental pollution, leaving harmful effects on living organisms [1]. Anthropogenic activities escalate the production of air pollutants and soil contamination, and their devastating impacts on plant life and the ecosystem have garnered significant attention from the scientific community [2]. Any physical, chemical, or biological factor that affects the natural characteristics of indoor or outdoor environments is defined as air pollution. Some common air pollutant examples are ozone, nitrogen oxides, sulfur dioxide, and particulate matter that directly disrupt physiological processes (photosynthesis and respiration) in plants, leading to reduced growth and yield [3]. Similarly, industrial activities accumulate heavy metals in soil, such as cadmium, lead, arsenic, chromium (Cr), copper (Cu), and mercury, posing severe threats to plant development by inducing oxidative stress, reducing root development, disrupting nutrient uptake, stunted growth, and impairing cellular functions [4]. Heavy metal in soil is taken up by roots and accumulated in the vegetative tissues and seeds, and can enter directly into the animal and human body upon consumption, causing significant hazards for living organisms, thus disrupting the entire ecosystem [5]. Under natural environments, air pollution and heavy metal stress coexist near industrial and urban areas, creating a tough situation for plants, where they have to counter combined adverse conditions simultaneously [6]. Understanding how plants regulate transcription factor genes to combat individual or combined stress conditions is crucial to developing strategies to enhance plant hardiness against hazardous materials [7].

Transcription factor (TF) genes play crucial roles in plant responses to hazardous pollutants [7,8]. These TFs control the expression of downstream stress-responsive genes and modulate complex signaling networks at the cellular level that enable plants to adapt and survive under challenging conditions [9]. Several TF families, such as NAC, CCAAT-DR1, HB, ARID, AREB/ABF, CCAAT-HAP5, C2C2-Dof, C2C2-CO-like, E2F-DP, ABI3VP1, C2C2-Gata, WRKY, DREB1/CBF, C2C2-YABBY, ARF, AtSR, bHLH, MYB, CPP, E2F-DP, MYC, HSF, bZIP, EMF1, MADS, TUB, C2H2, C3H, CCAAT-HAP2, AP2/ERF, and CCAAT-HAP3SBP, etc., have been well-known in plant responses to stresses [7,8,9,10]. These TFs activate or repress the expression of target genes that help plants to adapt to environmental challenges. Among these TFs, the MYB, WRKY, NAC, bHLH, and bZIP have emerged as key players in regulating stress responses against hazardous air pollutants. These TFs regulate a wide range of biological processes, including antioxidant defense, metal ion homeostasis, polyphenols, regulation of stress-responsive genes, and signaling pathways under environmental stress [7,9,10]. MYB TFs are known to modulate the downstream genes involved in flavonoid biosynthesis, which play a protective role against oxidative stress induced by air pollutants [11]. WRKY transcription factors regulate the expression of genes involved in reactive oxygen species (ROS) scavenging, heavy metal chelation, and stress signaling pathways [8]. Similarly, NAC transcription factors modulate the expression of genes related to metal chelation, metal sequestration, metal transport, antioxidant defense, and are involved in cellular detoxification [7,11,12,13].

The bHLH and bZIP families of TFs also play significant roles in abiotic stress [14]. bHLH TFs, such as FIT and PYE, are essential for iron homeostasis and the regulation of metal uptake and distribution [14,15]. bZIP TFs, such as AREB/ABF and HY5, activate the antioxidant defense mechanisms under oxidative stress conditions. These TFs often function in interconnected networks, allowing for a coordinated response to individual or combined stresses. The bZIP and WRKY TFs enhance the expression of genes involved in detoxification pathways, providing a robust defense against both air pollutants and heavy metals [8,16]. Identification and understanding of the complex interplay between different TF families and their target genes is crucial for developing stress-tolerant crop varieties. The identification and characterization of novel TFs involved in stress responses have opened new avenues for genetic engineering approaches aimed at enhancing plant tolerance to environmental pollutants [10].

In this review, we synthesize current knowledge on the molecular and metabolic strategies plants employ to counteract hazardous air pollutant stress. We highlight the pivotal roles of transcription factors (e.g., MYB, WRKY, NAC) in orchestrating stress-responsive gene networks, alongside the interplay of MAPK and phytohormone signaling cascades (e.g., SA, JA, ethylene) in mitigating oxidative damage. Furthermore, we explore how secondary metabolites—flavonoids, lignin, and terpenoids—act as frontline antioxidants to neutralize ROS and detoxify pollutants. By integrating recent advances in transcriptomics and metabolomics, this review underscores the potential of genetic engineering to develop resilient crops, while identifying critical gaps in understanding plant adaptation to combined stressors. Our aim is to provide a comprehensive resource for advancing research and applications in sustainable agriculture under escalating environmental pollution.

## 2. Anthropogenic and Natural Sources of Air Pollutants: Impacts on Plant Systems

**Figure 1:**
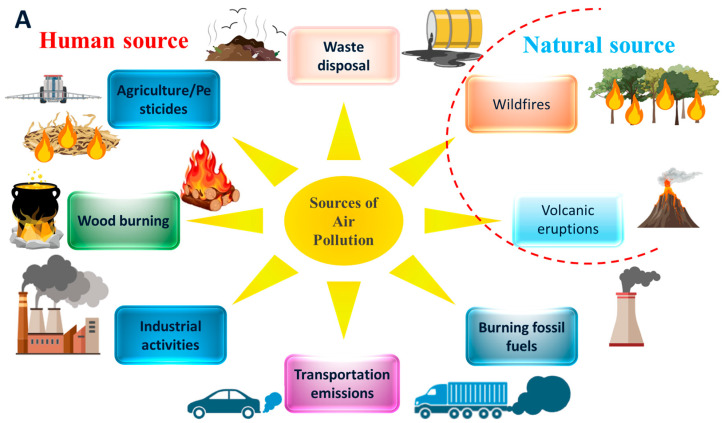

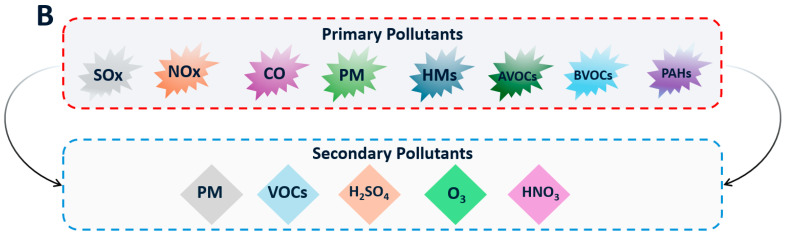
Human and natural sources of air pollution. (**A**) Major sources of air pollution, categorized into human activities and natural processes. Human activities include agriculture (e.g., pesticide use), industrial activities, transportation emissions, waste disposal, and wood burning. Natural sources encompass wildfires, volcanic eruptions, and biogenic emissions. (**B**) Classification of air pollutants into primary and secondary types. Primary pollutants are directly emitted into the atmosphere and include sulfur oxides (SOx), nitrogen oxides (NOx), carbon monoxide (CO), particulate matter (PM), heavy metals (HMs), volatile organic compounds (VOCs), biogenic volatile organic compounds (BVOCs), and polycyclic aromatic hydrocarbons (PAHs). Secondary pollutants are formed in the atmosphere through chemical reactions involving primary pollutants and include particulate matter (PM), ozone (O_3_), sulfuric acid (H_2_SO_4_), and nitric acid (HNO_3_).

The sources of air pollutants include a mixture of hazardous compounds from natural and anthropic activity (Figure 1). Industrial production, fossil fuel combustion, traffic and mobility, pesticide usage, heating systems, and burning of agricultural wastes are the key sources of air pollution, which release hazardous substances into the atmosphere [17,18]. These hazardous substances include primary pollutants (direct discharge into the environment) and secondary pollutants (formed by reaction between primary pollutants and other molecules in the atmosphere) (Figure 1) [19]. The sources of hazardous substances and different kinds of pollutants are represented in Figure 1. These all-hazardous air pollutants have significant effects on plants’ physiological processes, juvenile phase, and reproductivity, restricting growth and development and reducing yields [4,5,7,10,19].

### Particulate Matter (PM10 and PM2.5) and Its Impact on Plants

Particulate matter (PM), especially PM10 (≤10 µm) and PM2.5 (≤2.5 µm), is a major air pollutant that adversely affects plant physiology and molecular processes. PM deposits on leaf surfaces, clogging stomata and reducing photosynthetic efficiency, while finer particles (PM2.5) can penetrate tissues, inducing oxidative stress via reactive oxygen species (ROS) overproduction [20,21]. PM exposure upregulates stress-responsive genes, including those encoding for ROS-scavenging enzymes (e.g., SOD, CAT, APX) and transcription factors (e.g., WRKY, MYB, NAC) [3,4]. For instance, Arabidopsis exposed to PM2.5 shows elevated expression of RBOHD (Respiratory Burst Oxidase Homolog D), amplifying ROS signaling [20]. PM increases the activity of antioxidant enzymes (SOD, CAT, POD) and phenylpropanoid pathway enzymes (PAL, CHS), which are critical for detoxification [6,7]. Plants accumulate secondary metabolites (e.g., flavonoids, lignin, phenolic acids) to counteract PM-induced oxidative damage. For example, PM2.5 exposure in Triticum aestivum elevates rosmarinic acid and quercetin levels, which act as ROS scavengers [22]. PM often co-occurs with heavy metals (e.g., Pb, Cd adsorbed on particles), exacerbating toxicity. Plants employ chelators (e.g., glutathione, phytochelatins) and upregulate metal transporters to mitigate damage [21].

## 3. Signal Transduction Pathways Activated by Air Pollutants in Plants

**Figure 2:**
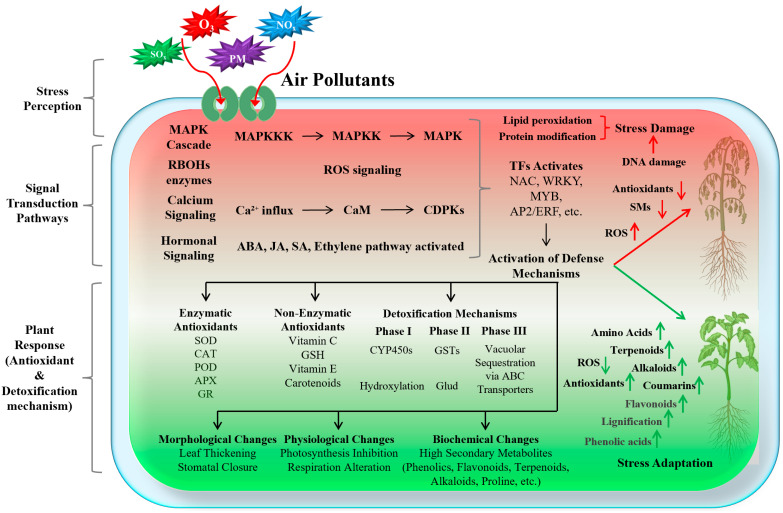
Air pollutant stress adaptation mechanism in plants. Abbreviations: RBOHs: Respiratory Burst Oxidase Homologs, CaM: Calmodulin, CDPKs: Calcium-Dependent Protein Kinases, TFs: Transcription Factors, SMs: Secondary Metabolites, SOD: Superoxide dismutase, CAT: Catalase, POD: Peroxidase, APX: Ascorbate peroxidase, GR: Glutathione reductase, GSH: Glutathione, ABC Transporters: ATP-Binding Cassette Transporters. The plant responses to air pollutant stress are categorized into (1) Stress perception: the air pollutants (SOx, NOx, O_3_, PM, etc.) passively enter leaf tissues through open stomata during normal gas exchange. These air pollutants react with the cellular components and generate ROS and activation of RBOHs, which amplify the stress signal. (2) The signal transduction pathways (MAPK cascade, calcium signaling, and hormonal signaling) are activated, leading to the induction of transcription factors (TFs) such as NAC, WRKY, MYB, AP2/ERF, etc. These TFs regulate the expression of downstream genes involved in the antioxidant defense system and detoxification mechanisms. (3) The plant responds through morphological changes, physiological changes, and biochemical changes (e.g., increased production of secondary metabolites like phenolics, flavonoids, lignification, terpenoids, alkaloids, etc.). These coordinated responses enable the plant to adapt to and mitigate the effects of air pollutant stress, whereas the plants that fail to activate such responses reveal lipid peroxidation, protein modification, and DNA damage, which causes severe stress damage or even cell/plant death.

Plants activate their signaling transduction mechanisms when exposed to different air pollutants. The air pollutant enters the plant leaf tissues via stomata [7,10]. These air pollutants, such as O_3_, NO_2_, etc., react with proteins and lead to protein modification, lipid peroxidation, and DNA damage (Figure 2). These alterations induce ROS overproduction, especially of superoxide (O_2_^−^) and hydrogen peroxide (H_2_O_2_). Under normal conditions, plants produce ROS during metabolic process; however, their excessive production causes dual functions: (1) oxidative damage to cellular components and (2) acting as a signaling molecule, which initiates a cascade of defense response that includes mitogen-activated protein kinase (MAPK) pathways, calcium signaling, and hormonal networks such as abscisic acid (ABA), jasmonic acid (JA), and ethylene [4,9,10]. The Respiratory Burst Oxidase Homologs (RBOHs) are subsequently activated through these calcium signals and MAPK-mediated phosphorylation, amplifying ROS production to regulate stress responses (Figure 2). These signaling pathways activate the regulatory transcription factors that modulate the expression of downstream stress-responsive genes, enabling plants to adapt to stress conditions [9].

### 3.1. MAPK Cascades in Plant Responses to Oxidative Stress

The MAPK cascade plays a pivotal role in plant responses to diverse abiotic stresses [23]. This signaling mechanism comprises three hierarchical protein kinases that operate through sequential phosphorylation events: MAPK kinase kinase (MEKK) activates MAPK kinase (MKK), which subsequently phosphorylates MAPK (MPK). Specific stress responses are mediated by distinct MAPK signaling modules, organized as MKK-MPK or MEKK-MKK-MPK units [24]. Upon ozone exposure, Arabidopsis MPK3 and MPK6 undergo rapid and transient activation (within 0.5–2 h). Following activation, these MPKs translocate to the nucleus and regulate expression of specific genes, including tobacco orthologs SIPK and WIPK, which exhibit ozone-induced expression patterns [25]. Interestingly, MPK3 and MPK6 display reciprocal regulation mechanisms—inhibition of MPK3 enhances MPK6 activation, while MPK6 suppression results in sustained and intensified MPK3 activity [26,27]. During pathogen stress responses, MKK4/MKK5 activation occurs via MEKK1 [28], and MKK5 also participates in MPK3/MPK6 activation following ozone pollutant exposure [29]. Appropriate ozone response requires precise calibration of MPK3/MPK6 activity levels, as prolonged activation or aberrant signal intensity increases ozone vulnerability, as shown by the ozone-sensitive radical-induced cell death1 (rcd1) mutant, which exhibits extended MPK3/MPK6 activation than wild-type plants [30]. Research indicates that MPK3/MPK6 substrates participate in transcriptional regulation, nitric oxide (NO) signaling networks, signaling pathways, and ethylene biosynthesis [31]. Additionally, the MKK4/MKK5–MPK3/MPK6 module crucially regulates stomatal dynamics, indirectly influencing ozone penetration into leaf tissues [27]. A significant knowledge gap remains regarding the specific mechanisms that enable a limited number of kinases to orchestrate such diverse stress responses.

### 3.2. Phytohormonal Crosstalk in Air Pollution Stress Adaptation

Phytohormones serve as crucial regulators for alleviating the adverse effects of environmental stresses in plants. Salicylic acid (SA), a versatile phenolic compound, plays integral roles in plant developmental processes and stress responses, including ozone exposure [32]. SA operates within interconnected signaling networks alongside other phytohormones such as jasmonic acid and ethylene when responding to air pollutant stress [10]. Research has demonstrated SA’s ability to induce stomatal closure, a key protective mechanism. JA similarly modulates stomatal aperture but typically promotes closure under oxidative stress, synergizing with SA to limit pollutant entry [33]. While SA signaling in guard cells, together with its crosstalk with other pathways, significantly contributes to stomatal immunity, the underlying molecular mechanisms remain incompletely characterized in response to air pollution [3,10].

SA biosynthesis in plants occurs via two primary pathways: the phenylpropanoid pathway and the isochorismate synthase pathway in plant species [34]. Phenylalanine ammonia-lyase (PAL) represents a critical enzyme in one SA biosynthetic route [34,35]. Studies have revealed that ethylene can modulate ozone-induced SA accumulation by altering the expression patterns of PAL and chorismate mutase in tobacco plants [36]. Under ozone stress, Arabidopsis ecotype Wassilewskija exhibits reduced expression of AtSR/NFκB transcription factor family members, C2-domain proteins, and genes involved in cell wall formation and critical point drying processes [37]. The RCD1 protein (radical-induced cell death 1) has been identified as a potential regulatory candidate in this response [37,38]. Investigations using 14C-labeled benzoic acid as a precursor revealed distinct pathway preferences between species: while tobacco primarily utilizes the phenylalanine pathway with minimal isochorismate synthase induction upon ozone exposure, Arabidopsis shows enhanced ICS activity [36]. This pathway distinction is further supported by observations in the salicylic acid induction-deficient 2 (sid2) Arabidopsis mutant, which lacks ICS1 activity and consequently exhibits reduced SA levels following ozone treatment [36,37,38]. These findings collectively show species-specific SA biosynthetic routes: predominantly via benzoic acid from phenylalanine in tobacco, while Arabidopsis primarily utilizes the isochorismate pathway.

Previous research has exhibited that SA accumulation exhibits a dose-dependent relationship with ozone-induced leaf lesion formation [39]. Tobacco cultivar ‘Xanthi’ expressing the NahG gene showed reduced SA accumulation and subsequently decreased lesion development during ozone exposure. Notably, both SA-deficient and hyperaccumulating genotypes show increased ozone sensitivity, suggesting an optimal SA threshold for defense [36,40]. In Arabidopsis, the ozone-tolerant Col-0 ecotype transformed with NahG exhibited enhanced sensitivity to ozone compared to wild-type plants, while the ozone-sensitive Cvi-0 ecotype accumulated excess SA (3× higher than Col-0) [36,40]. This implies that balanced SA levels—not merely their presence—mediate ozone tolerance, potentially by modulating PR1 expression and redox homeostasis. Ozone exposure significantly upregulated PR1 (AT2G14610) expression in Cvi-0 plants, with expression levels surpassing those observed in Col-0 even under control conditions [37,38].

In the ethylene biosynthesis pathway, two enzymes play pivotal roles: ACC synthase (ACS) and ACC oxidase (ACO) [41]. The process begins with the conversion of S-adenosyl-L-methionine to 1-aminocyclopropane-1-carboxylic acid (ACC) by ACS, followed by oxidative cleavage catalyzed by ACO to generate ethylene [42]. These enzymatic activities are encoded by conserved gene families across diverse organisms, all performing identical biochemical functions in the ethylene production cascade [41]. Among the nine characterized ACS genes in Arabidopsis, only one appears to function as the primary regulator triggering ethylene levels during ozone exposure. Notably, a consistent relationship exists between ozone-induced ethylene production rates and the severity of leaf damage across multiple plant species [37]. For instance, the ozone-tolerant ‘Bel-B’ tobacco cultivar generated significantly less ethylene compared to its ozone-sensitive counterpart ‘Bel-W3′ when subjected to ozone stress. Furthermore, the application of ethylene biosynthesis inhibitors during ozone exposure effectively mitigated leaf injury [43]. These observations suggest that increased ozone-induced foliar damage associated with ethylene might result from the generation of harmful free radicals and toxic aldehydes through direct chemical interactions between ozone and ethylene molecules [43,44]. Notably, certain plant-associated microbes expressing ACC deaminase can mitigate this damage by cleaving the ethylene precursor 1-aminocyclopropane-1-carboxylic acid (ACC), thereby reducing stress ethylene levels and enhancing pollutant tolerance [45,46,47]. Mechanistically, ethylene functions as a signaling molecule through receptor binding, and the evidence presented indicates that ethylene signaling pathways become activated in response to ozone, ultimately contributing to leaf damage manifestation.

JA plays a critical role in mitigating ROS-mediated lesion formation in ozone-exposed leaf tissue. Research utilizing ozone-sensitive JA-deficient Arabidopsis mutants has revealed significant insights into this protective mechanism [48]. Several JA-related mutants demonstrate heightened ozone sensitivity, including the JA-insensitive 1 (oji1), coronatine insensitive1 (coi1), methyl jasmonate-resistant 1 (jar1), and 12-oxophytodienoate reductase 3 (opr3) mutants. Experimental evidence indicates that methyl jasmonate (MeJA) pretreatment significantly enhances ozone tolerance in both Arabidopsis and tobacco species, strongly suggesting JA’s functional involvement in ozone stress response pathways [48]. Mechanistically, MeJA enhances expression of genes encoding ethylene response sensors (ERS2), which effectively inhibit foliar damage [49]. JA appears to counteract ozone-induced ethylene-dependent lesion development [37,49]. Furthermore, JA likely facilitates antioxidant production, providing an additional protective mechanism against ozone damage. MeJA treatment has been demonstrated to enhance expression of genes associated with the ascorbic acid pathway and glutathione synthesis enzymes [50,51,52]. These findings collectively suggest that JA-mediated antioxidant accumulation represents a significant mechanism underlying increased ozone tolerance in plants.

### 3.3. Integrated Phytohormone Networks Under Stress Conditions

Classical phytohormones (ethylene, abscisic acid, auxins, and cytokinins) and signaling molecules (salicylic acid, jasmonic acid, proline, and brassinosteroids) exhibit different responses to chronic and acute air pollutant exposure [33]. The air pollutants trigger ROS accumulation within cellular compartments, ultimately leading to programmed cell death (PCD), which operates under a regulatory mechanism involving feedback inhibition [27]. Recent investigations have increasingly focused on elucidating phytohormone interactions under ozone stress conditions in plant systems [53]. Evidence suggests that ozone exposure initiates ROS generation, subsequently triggering the production of ethylene, salicylic acid, and jasmonic acid concurrent with PCD activation. Within this cascade, ethylene accumulation appears to be essential for sustaining continuous ROS production [27,53].

During oxidative cell death processes, phytohormones exhibit complex antagonistic interactions. JA, SA, and ethylene (ET) demonstrate regulatory crosstalk, while ABA plays a crucial role in stomatal regulation to restrict pollutant entry and appears to counteract ET-mediated acceleration of cell death [54]. ABA is essential for regulating developmental processes and gene expressions related to stomatal function [55]. Studies have established that ABA mediates drought stress responses by promoting stomatal closure [55,56]. Interestingly, ozone-induced ET accumulation appears to interfere with ABA-mediated stomatal signaling in ozone-sensitive species, potentially promoting stomatal opening [27,53]. This ET accumulation can effectively disrupt ABA-mediated stomatal regulation pathways [57]. Research has further shown that ROS signaling activates auxin responses through complex mechanisms [58]. Studies utilizing ozone as a model ROS inducer revealed that apoplastic ROS altered auxin homeostasis and signaling pathways, thereby modulating gene expression patterns in Arabidopsis [38]. Limited research exists regarding cytokinins’ (CKs) protective role against oxidative damage following ozone exposure [56]. However, in plants, kinetin has been shown to delay leaf senescence, maintain free sterol levels, and inhibit foliar necrosis during stress [45,46,47].

## 4. Enzymatic and Non-Enzymatic Antioxidant Systems Under Combined Pollution and Heavy Metal Stress

A critical outcome of the signaling transduction cascade is the activation of the plant’s antioxidant defense system, which includes both enzymatic components (e.g., superoxide dismutase, catalase, and peroxidases) and non-enzymatic components (e.g., ascorbate, glutathione, and tocopherols) [59]. These antioxidants work synergistically to scavenge excess ROS, preventing oxidative damage to cellular components such as proteins, lipids, and DNA [11,59]. Concurrently, detoxification mechanisms are engaged to neutralize pollutants and their byproducts through three sequential phases: Phase I (Functionalization): Cytochrome P450 enzymes (e.g., CYP450s) oxidize pollutants to increase reactivity. Phase II (Conjugation): Glutathione S-transferases (GSTs) or glycosyltransferases link activated pollutants to glutathione/sugars for solubilization. Phase III (Compartmentalization): ATP-binding cassette (ABC) transporters sequester conjugated metabolites into vacuoles or apoplast [60,61]. The efficiency of these defense systems varies considerably among species and cultivars, contributing to differential pollution sensitivity [62]. Despite significant advances in understanding plant responses to air pollution, substantial knowledge gaps remain regarding the precise molecular mechanisms through which ROS and RBOHs orchestrate signal transduction under pollutant stress [10,62]. The complexity of these interactions is further compounded by the influence of other environmental factors, such as temperature, light intensity, and water availability [11], which can substantially modify plant responses to air pollution [62].

Plant exposure to atmospheric heavy metals (HMs) also induces ROS overproduction, leading to oxidative damage, lipid peroxidation, DNA damage, and disruptions in calcium homeostasis [62]. In response to HM stress, plants enhance the activity of antioxidant enzymes such as catalase (CAT), superoxide dismutase (SOD), glutathione peroxidase (GPX), and ascorbate peroxidase (APX). SOD, a key antioxidant enzyme, is present in most subcellular compartments and catalyzes the dismutation of superoxide radicals (O_2_^•−^) into oxygen and hydrogen peroxide (H_2_O_2_) [63]. SOD, located in various subcellular compartments, represents a primary defense mechanism against oxidative damage; it catalyzes the conversion of superoxide radicals (O_2_^−^) to oxygen and hydrogen peroxide [64]. In chloroplasts, the ascorbate-glutathione cycle plays a crucial role in detoxifying H_2_O_2_, with enzymes like dehydroascorbate reductase (DHAR), monodehydroascorbate reductase (MDHAR), and glutathione reductase (GR) participating in this process [65]. CAT, localized in peroxisomes, dismutates H_2_O_2_ into water and oxygen, while APX modulates H_2_O_2_ for signaling purposes. Studies have shown that antioxidant enzyme activities increase under HM stress, with elevated SOD, CAT, and GPX activities observed in response to lead (Pb) and arsenic (As) exposure [66]. Non-enzymatic antioxidants, such as proline, ascorbate, and glutathione, also accumulate under stress conditions, further enhancing plant resilience [11,59,67]. Overexpression of antioxidant genes, such as *TaCAT3* in wheat, has been shown to confer tolerance to As stress, highlighting the potential for biotechnological interventions to improve plant stress tolerance [68]. These findings collectively emphasize the critical role of antioxidant defenses in mitigating the adverse effects of air pollutants and HMs on plants.

Several studies have highlighted the potential role of antioxidants in mitigating HM stress in plants [59,63]. Research has shown that the activities of key antioxidant enzymes—such as SOD, GPX, CAT, and APX—increase significantly with rising lead (Pb) concentrations, particularly within the range of 50 to 100 μM [69]. Comparative analyses of Pb-stress tolerance across flax genotypes revealed that the ‘Milas’ variety exhibited superior SOD and peroxidase (POD) activity compared to other genotypes [70]. Similarly, exposure to As has been shown to enhance SOD and CAT activity in plants [71]. In cadmium (Cd)-stressed *Brassica juncea* cultivars, elevated levels of non-enzymatic antioxidants—including proline, ascorbate, and glutathione—were observed, along with increased activity of enzymatic antioxidants such as SOD, CAT, glutathione S-transferase (GST), GR, APX, and POX [72]. Furthermore, transgenic approaches have underscored the importance of catalase in HM stress tolerance. Overexpression of *Triticum aestivum* CAT (*TaCAT3*) conferred enhanced As stress tolerance, while transgenic lines expressing *TaCAT3-B* gene exhibited improved resistance to both arsenite (AsIII) and arsenate (AsV) toxicity [68]. These studies have shown the critical function of antioxidants in mitigating HM stress in plants.

## 5. Transcriptomic Reprogramming Under Air Pollution Stress

Transcriptomic analyses of *Arabidopsis* under ozone (O_3_) stress have unveiled ecotype-specific responses, underscoring the complexity of transcriptional regulation in plant defense mechanisms. For instance, the Cvi-0 ecotype exhibited upregulation of three ethylene-responsive genes (*AT1G49830*, *AT1G55150*, and *AT2G22300*) with undetermined functions, while both Cvi-0 and Col-0 ecotypes showed downregulation of *At5g44440* and *At2g26020* genes during O_3_ exposure [37]. Similar transcriptional patterns were observed in other crop species, such as potato and tomato, where O_3_ exposure induced the expression of ethylene biosynthesis genes (e.g., *ST-ACS4*, *ST-ACS5*, *LE-ACS1A*, *LE-ACS2*, and *LE-ACS6*) [73]. JA appears to counteract O_3_-induced ethylene-dependent lesion development, with JA-induced genes like AT2G24850 and AT5G24770 playing a protective role. Additionally, MeJA enhances the expression of genes involved in key antioxidant systems, including components of the ascorbic acid pathway (*VTC1*, *VTC2*, *DHAR*, and *MDHAR*) and glutathione synthesis enzymes (GSH1 and GSH2) [50,51,52]. These findings highlight the intricate interplay between hormonal signaling and transcriptional regulation in mitigating oxidative stress caused by air pollutants.

Urban areas face growing air quality concerns due to elevated nitrogen dioxide (NO_2_) emissions from human activities [10]. Despite this, research exploring NO_2_’s impact on plant development remains limited, with most studies focusing on injury symptoms, physiological responses, and photosynthetic efficiency. A comprehensive investigation of air pollution effects on plant gene expression examined approximately 372 *Arabidopsis thaliana* accessions exposed to NO_2_ (up to 30 ppm for 1 h) or O_3_ (up to 400 ppm for 2–6 h). Genome-wide association studies (GWAS) revealed varying tolerance levels among accessions [74]. Transcriptomic and microarray analyses demonstrated that both pollutants elicited similar molecular responses, particularly involving hormone signaling pathways. Expression of JA and ethylene signaling marker genes, such as those cooperatively regulated by ethylene and jasmonate 1 (CEJ1), and SA signaling genes, including glutaredoxin 480 (GRX480) and flavin-dependent monooxygenase 1 (FMO1), showed significant upregulation in NO_2_- and O_3_-exposed plants compared to controls [74]. These findings emphasize the critical role of transcriptional regulation in mediating plant responses to air pollutants.

Further transcriptomic analysis of *Ambrosia artemisiifolia* L. (common ragweed) pollen subjected to extended fumigation (61 days) with varying concentrations of NO_2_ (40 ppb control, 80 ppb treatment) and O_3_ (40 ppb control, 80 ppb, and 120 ppb treatments) revealed enrichment of gene ontology (GO) terms associated with abiotic and biotic stress responses, JA biosynthesis, and phosphate homeostasis [75]. Additionally, GO terms related to ethylene perception, ABA, and auxin signaling pathways were predominantly enriched in upregulated transcripts from treated pollen [75]. These results underscore the critical role of phytohormones in pollution response mechanisms, with JA signaling emerging as particularly important in mediating plant responses to both NO_2_ and O_3_ exposure. Differential responses to NO_2_ and O_3_ exposure were observed in genes regulating ROS production and metabolism. O_3_ treatment upregulated respiratory burst oxidase homolog F (RBOH), which encodes an NADPH oxidase essential for ROS synthesis, while NO_2_ exposure resulted in its downregulation [74]. Nevertheless, substantial evidence confirms NO_2_-induced oxidative stress. In a recent investigation, *Bougainvillea spectabilis* Willd. seedlings subjected to acute high-concentration NO_2_ fumigation (8 µL·L^−1^ for 8 h) developed characteristic yellow-brown leaf spotting indicative of oxidative damage [76]. Biochemical analysis revealed significantly elevated activities of POD, SOD, and CAT in NO_2_-treated seedlings compared to controls, demonstrating activation of antioxidative defense mechanisms [76]. Furthermore, comprehensive metabolomic profiling identified substantial alterations in pathways related to flavonoid and stilbene biosynthesis, amino acid metabolism, and tricarboxylic acid (TCA) cycle intermediates between treated and control plants, providing additional evidence for NO_2_-induced oxidative stress in *B. spectabilis* [76].

**Figure 3:**
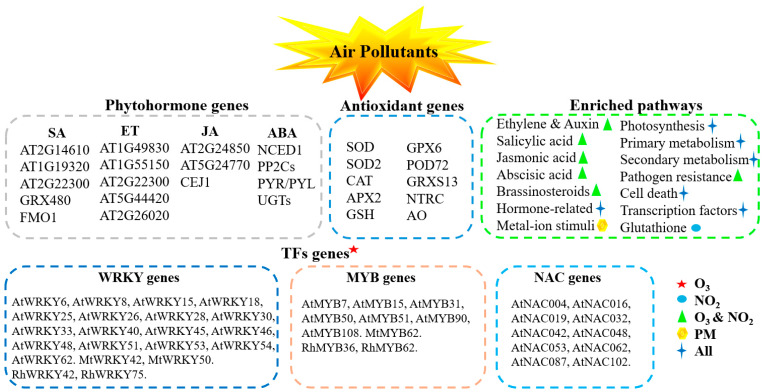
Molecular response of plants under different air pollutant stress. Abbreviations: SA: Salicylic Acid, ET: Ethylene, JA: Jasmonic Acid, ABA: Abscisic Acid, SOD: Superoxide dismutase, GPX6: Glutathione Peroxidase 6, SOD2: Superoxide dismutase 2, POD72: Peroxidase 72, CAT: Catalase, GRXS13: Glutaredoxin 13, GRX480: Glutaredoxin 480, FMO1: Flavin-dependent monooxygenase 1, APX2: Ascorbate peroxidase 2, NTRC: NADPH Thioredoxin reductase C, GSH: Glutathione, AO: Ascorbate oxidase, Rh: *Rosa hybrida* (L.) [77], Mt: *Medicago truncatula* [78], At: *Arabidopsis thaliana*. Plants activate a complex network of molecular pathways in response to air pollutants such as ozone (O_3_), nitrogen dioxide (NO_2_), and particulate matter (PM). The enrichment of various pathways, including ethylene, auxin, salicylic acid, jasmonic acid, abscisic acid, brassinosteroids, and glutathione metabolism, which are critical for stress adaptation.

Plants activate a complex network of molecular pathways and genes in response to different air pollutant stresses (Figure 3). Key genes associated with phytohormone signaling (e.g., SA, ET, JA, ABA), antioxidant defense mechanisms (e.g., SOD, CAT, APX, GSH), and transcription factors (e.g., WRKY, MYB, NAC) are upregulated to mitigate oxidative stress induced by air pollutants. This molecular reprogramming highlights the intricate interplay between hormonal signaling, antioxidant systems, and transcriptional regulation in plant stress responses (Figure 3). Additionally, plants exhibit enhanced production of secondary metabolites and activation of detoxification mechanisms to counteract the harmful effects of air pollutants. These findings collectively highlight the critical role of transcriptional regulation in plant responses to air pollutants, offering insights into potential strategies for enhancing stress tolerance.

## 6. Metabolic Adaptations and Secondary Metabolite Production in Polluted Environments

Secondary metabolites in plants are predominantly regulated by genetic factors in response to environmental stimuli, exhibiting high species specificity [11]. These bioactive compounds are synthesized via specialized metabolic pathways as defensive mechanisms when plants encounter stressful conditions [59]. The major secondary metabolites originate from three principal biosynthetic routes: phenylpropanoid, isoprenoid, and alkaloid pathways [79]. Beyond their well-recognized antimicrobial properties, these phytochemicals demonstrate remarkable antioxidant capacity, effectively neutralizing ROS generated by oxidative stress [11,59]. The significant ecological and economic importance of these compounds has driven extensive research into their biosynthetic pathways, including associated enzymes and regulatory genes [80,81,82]. Current evidence suggests that metabolite biosynthesis typically exhibits compartmentalization at both inter- and intracellular levels, necessitating sophisticated trafficking mechanisms for intermediate and end products within and between cells [83]. Additionally, numerous secondary metabolites are stored in inactive forms or specific compartments, requiring activation or release from these compartments to exert their biological functions [84,85].

### Phenylpropanoid Metabolism and Its Role in Pollutant Detoxification

**Figure 4:**
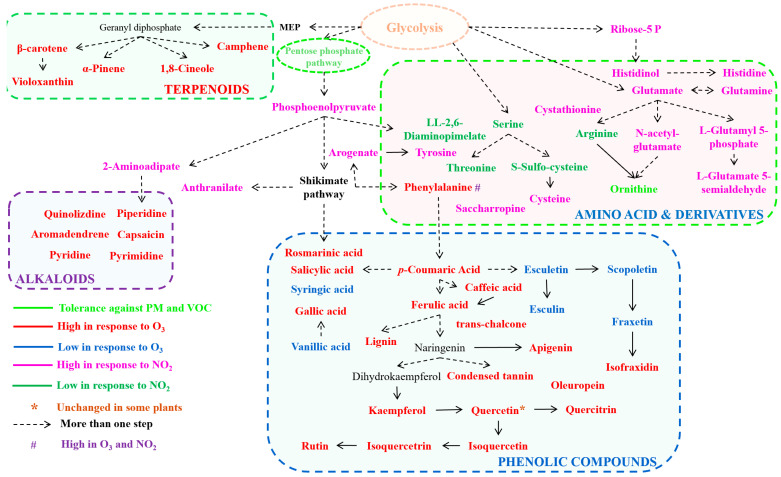
Plant metabolic responses to different air pollutants. The figure showed the metabolic adaptations of plants in response to various air pollutants, including particulate matter (PM), volatile organic compounds (VOCs), ozone (O_3_), and nitrogen dioxide (NO_2_). Plants exhibit differential metabolic responses depending on the type and concentration of pollutants.

Plants exhibit complex metabolic adaptations in response to various air pollutants (Figure 4). These air pollutants trigger distinct metabolic pathways, leading to the production of specific compounds that help plants mitigate stress and maintain cellular homeostasis (Figure 4). These bioactive compounds may act as protective agents, helping plants detoxify harmful pollutants, neutralize the ROS produced during oxidative stress induced by air pollutants, and ultimately reduce oxidative damage [11,59].

Phenolic compounds in plants predominantly originate from phenylpropanoid metabolism, a pathway evolutionarily significant for enabling the plant transition from aquatic to terrestrial environments. The fundamental C6-C3 skeletal structure characterizes all phenylalanine-derived compounds (Figure 4). Contemporary research has consistently demonstrated that plants under ozone stress exhibit upregulation of secondary metabolites, particularly within the shikimate and flavonol biosynthetic pathways [86]. The biosynthesis of flavonoids, stilbenes, hydroxycinnamates, and phenolic acids involves a sophisticated network of interconnected metabolic pathways, primarily centered around shikimate, phenylpropanoid, and flavonoid biosynthesis [84,86]. Each distinct branch of shikimic acid metabolism contributes specialized phenolic compounds to the overall metabolite profile. The enzymes PAL and tyrosine ammonia-lyase (TAL) catalyze critical conversion reactions: phenylalanine to cinnamic acid and tyrosine to p-coumaric acid, respectively [35]. PAL activity is frequently utilized as a molecular biomarker indicating activation of plant defense mechanisms, encompassing the production of both protective and structural compounds.

The interrelationship between O_3_ stress and phenylpropanoid biosynthesis has been extensively characterized through contemporary and historical phytochemical investigations [87,88]. However, the specific cellular localization of these biosynthetic processes remains insufficiently documented. The vacuolar sequestration of phenylpropanoids is extensively documented; these compounds predominantly enter vacuoles as glycosides or alternative conjugated forms. The translocation mechanism appears potentially contingent upon terminal conjugation reactions. Several enzymes participating in the general phenylpropanoid pathway, particularly PAL and CHS, exhibit cytoplasmic distribution [11]. Furthermore, substantial evidence indicates that cytochrome P-450 enzymes (including cinnamate-4-hydroxylase, isoflavone synthase, and isoflavone-2′-hydroxylase) function as transmembrane proteins associated with the endoplasmic reticulum or affiliated cellular components [81,89].

Upon entry into plant tissues, ozone first encounters resistance in the apoplastic region, comprising the cell wall and extracellular fluid [90]. While ascorbic acid within the apoplastic fluid provides initial protection against ozone-generated ROS, numerous studies have documented elevated levels of phenolic compounds in response to ozone exposure [84], suggesting their potential involvement in ROS neutralization (Figure 4). Although Gupta and De (2017) have proposed theoretical mechanisms for cell wall-bound phenolics as ROS scavengers, their specific role in ozone detoxification remains uncertain [91]. Research has demonstrated concentration-dependent ozone-stimulated lignin synthesis in foliar and stem cell walls, indicating a possible function in ROS neutralization [60,61]. The biosynthesis of lignin is connected to primary metabolism through the phenylpropanoid pathway. In dicotyledonous plants, phenylalanine serves as the fundamental precursor for all monolignols present in lignin and is derived from the shikimate pathway. Although phenylalanine synthesis primarily occurs within plastids, its subsequent transport to the cytosol via specialized cationic-amino acid transporters highlights the compartmentalized nature of phenylpropanoid metabolism [92].

The specific metabolic pathways and compounds involved in plant responses to air pollution showed that glycolysis and phosphoenolpyruvate pathways are activated to produce a diverse group of bioactive compounds and highlighting their importance in energy production and stress adaptation. Additionally, amino acid derivatives, including arginine and specialized metabolites like N-acetyl-glutamate and L-glutamate S-nondeldehyde, serve as key players in nitrogen metabolism and detoxification processes [62]. These compounds increased in plants in response to NO_2_ stress, except arginine, which was reduced (Figure 4). Phenolic acids such as salicylic acid and rosmarinic acid increased, while syringic acid and vanillic acid decreased under ozone stress in plants, suggesting their role in antioxidant defense and signaling during stress [60,93]. Furthermore, some alkaloids, terpenoids, polyamines (e.g., putrescine, spermidine) [94], and other secondary metabolites were also increased in response to air pollutants, which accumulated in plants as an adaptive strategy to mitigate the oxidative stress caused by pollutants [84]. Polyamines in particular stabilize membranes and scavenge ROS through their cationic nature, while also modulating antioxidant enzyme activity under stress [95]. These metabolic responses also contribute to their ability to maintain growth and productivity under challenging environmental conditions (Figure 4). Overall, Figure 4 provides a comprehensive overview of the metabolic flexibility of plants in adapting to air pollution, highlighting the interplay between primary and secondary metabolism in stress tolerance.

Flavonoids exhibit diverse cellular distribution, including vacuoles of mesophyll cells, chloroplasts, glandular trichomes, and the vacuoles and cell walls of epidermal tissues [96,97]. Notably, chloroplasts function not only as sites for flavonoid biosynthesis but also as significant accumulation reservoirs [96]. This strategic positioning, proximal to or within ROS generation sites, enables flavonoids to effectively attenuate photooxidative damage [59]. While flavonoid synthesis occurs primarily in the cytosol, these compounds are subsequently sequestered in vacuoles and demonstrate capacity for systemic transport throughout the plant [96,98]. This subcellular localization pattern strongly supports their proposed function as ROS scavengers. Research indicates that vacuolar flavonols in both mesophyll and epidermal cells serve as electron donors to phenol peroxidases (POX), thereby facilitating hydrogen peroxide detoxification within the organelle. Additionally, chloroplast-localized flavanols demonstrate efficacy in quenching singlet oxygen species [11,59,96,99].

Mitochondria exhibit greater susceptibility to ozone damage compared to chloroplasts, despite possessing a comprehensive array of antioxidant defense mechanisms [100]. Interestingly, the synthesis and accumulation of phenolic compounds within mitochondria remain largely unexplored, although these metabolites are recognized for their inhibitory effects on respiratory processes. In addition to aerial plant tissues, subterranean structures also synthesize and release modest quantities of flavonoids. For example, flavonoid aglycons (non-glycosylated flavonoids) exuded from leguminous root systems potentially function as signaling molecules essential for establishing symbiotic relationships with rhizobial communities in soil [62,84,90]. This suggests the possible enhancement of flavonoid exudation into the rhizosphere under ozone stress conditions.

## 7. Secondary Metabolites as Antioxidants and Chelators to Mitigate Oxidative Stress

In plant defense mechanisms against oxidative stress, flavonoids (including anthocyanins) serve as critical non-enzymatic antioxidants within the phenolic class of secondary metabolites [11,59]. Research by Tonelli et al. (2015) showed that *Melissa officinalis* shoot cultures, when exposed to O_3_, activated enzymes involved in phenolic metabolism, resulting in enhanced polymerization of cinnamyl alcohols (CAD), which maintained plant vitality [60]. Furthermore, differential responses were observed between cultivars under O_3_ stress conditions. The tolerant cultivar Kharchiya 65 exhibited superior oxidative stress tolerance through significantly elevated activities of CAD, 4CL, PAL, and chalcone isomerase (CHI), along with increased alpha-tocopherol accumulation. Conversely, the sensitive cultivar HD 2987 predominantly induced flavonoid biosynthesis as a mechanism to neutralize excessive ROS production under O_3_ stress [101]. Consistent with these findings, Pellegrini et al. (2018) documented that *Liriodendron tulipifera* displayed markedly increased PAL activity following a 45-day exposure to 120 ppb O_3_ [102]. This metabolic adaptation represents a strategic response to O_3_-induced oxidative damage, characterized by enhanced production of defensive secondary metabolites (specifically rutin and caffeic acid) and upregulated transcriptional expression of CHS and PAL genes.

The impact of elevated O_3_ concentrations on woody plants has predominantly been investigated through the lens of phenylpropanoid metabolism. These compounds serve critical functions in plant stress tolerance through their protective barrier formation and antioxidant properties [103]. The phenylpropanoid pathway, including its genes, enzymes, and metabolites, represents one of the primary targets of O_3_ exposure. Multiple studies with woody species have documented increases in total phenolic content following O_3_ treatment [104]. Research by Cotrozzi et al. (2018) revealed enhanced accumulation of isofraxidin and transchalcone in *Fraxinus excelsior* (European ash) when subjected to 150 ppb O_3_ for 8 h daily (Table 1), suggesting these compounds function in ROS detoxification mechanisms [105]. In Brazil’s early secondary succession species, *Astronium graveolens*, which exhibits high light sensitivity, researchers examined the combined effects of elevated light intensity and O_3_. This investigation demonstrated that polyphenols accumulated within the vacuoles of palisade parenchyma cells throughout the leaf blade, representing a coordinated defense response against these environmental stressors [104].

Polyphenols and their glycosylated derivatives function as potent antioxidants by directly neutralizing ROS and reactive nitrogen species (RNS) or by acting as peroxyl radical scavengers [11]. Notably, polyphenols containing two adjacent hydroxyl groups demonstrate metal chelation capabilities against transition metals [116]. Within the phenylpropanoid family, quercetin plays a particularly significant role in enhancing scavenging efficiency. The extensive cellular distribution of secondary metabolites, coupled with the quantity and positioning of hydroxyl groups on their ring structures, enables flavonoids to demonstrate superior ROS scavenging capacity compared to other phenylpropanoids [11,129]. Specifically, flavonoids with dihydroxy groups on the B-ring exhibit enhanced antioxidant activity. Through single electron oxidation reactions, flavonoids effectively reduce free radicals by transferring protons from their A and/or B rings, thereby generating less reactive flavonoid radicals [96]. Additionally, flavonoids inhibit oxygen radical formation by suppressing the enzymatic activities of cyclooxygenase, lipoxygenase, xanthine oxidase, and glutathione S-transferase [130]. In experimental conditions using Open-Top Chambers (OTCs), *Tibouchina pulchra* (Cham.) saplings exposed to ambient non-filtered air (NF) and NF supplemented with 40 ppb ozone (NF+O_3_) for 8-h daily periods demonstrated elevated flavonoid concentrations compared to those in carbon-filtered air (CF) environments [131].

Research demonstrates that exposure to elevated ozone concentrations induces increased lignin accumulation in the aboveground portions of multiple forage and cereal species [132]. While lignin deposition typically occurs in root endodermal and vascular cells under normal conditions, ozone preferentially triggers lignification in aerial tissues (e.g., leaves, stems) as a physical barrier against oxidative damage [94]. This response activates genes and enzymes involved in lignin biosynthesis, particularly PAL, as part of defensive mechanisms limiting cellular damage. PAL activation serves as a biochemical indicator of triggered defense responses, including the development of structural barriers and protective compounds. The enhanced PAL activity correlates with elevated lignin content in ozone-exposed foliar tissues, which differs compositionally from lignin in control plants [103]. Scientific literature consistently reports that ozone exposure not only increases lignin content but also alters its composition in affected plants [84]. For example, Betz et al. (2009) documented that following ozone exposure, *Fagus sylvatica* L. exhibited increased guaiacyl (G) and p-hydroxyphenyl (H) units, while syringyl (S) monomer content decreased [133]. Moreover, plants under ozone stress demonstrate lignin-adaptive mechanisms to maintain mechanical integrity under challenging conditions [133]. Investigations by Richet et al. (2012) revealed reduced cellulose-to-lignin ratios in ozone-treated hybrid poplar stems, suggesting prioritization of lignification over cellulose production under ozone stress, thereby promoting radial development [103]. This adaptive response potentially enables ozone-affected trees to sustain growth while optimizing carbon resource allocation. Numerous tree species exhibit alterations in their proanthocyanidin profiles in response to ozone exposure, as documented in various studies [133]. However, the scientific literature has predominantly investigated proanthocyanidins through the lens of their antioxidative capabilities, while largely neglecting their important structural and supportive functions within plant tissues.

Oxidative stress occurs when ROS accumulation exceeds the plant’s detoxification capacity, leading to cellular damage [134]. Research indicates that insufficient upregulation of phenylpropanoid metabolism and subsequent inadequate phenolic concentrations failed to mitigate ozone-induced H_2_O_2_ accumulation in poplar clone Eridano, resulting in oxidative damage [135]. Similarly, *Ginkgo biloba* exhibited reduced ozone stress tolerance when leaf phenolic concentrations decreased [136]. Interestingly, following ozone exposure, *G. biloba* leaves demonstrated enhanced antioxidant capacity through increased terpene synthesis. Terpenoids contribute to photorespiratory mechanisms that protect plants against oxidative stress and photodamage [137]. Carotenoids represent the most extensively studied terpenoids involved in photoprotection, with numerous studies documenting their modulation by ozone exposure [138]. Certain isoprenoids, including zeaxanthin and tocopherols, exhibit direct ROS scavenging capabilities through interactions with hydroxyl radicals [139]. Furthermore, investigations have revealed that isoprene contributes to cellular membrane stabilization, particularly the thylakoid membranes within chloroplasts [140,141].

Lignin biosynthesis exhibits notable alterations in response to O_3_ exposure. Experimental studies have demonstrated that both poplar leaves subjected to O_3_ stress display increased lignin concentrations due to enhanced activity within the lignin biosynthetic pathway [103]. The O_3_-induced lignins appear to serve a protective function by mitigating ROS propagation [142]. Evidence suggests these stress-induced lignins may function as antioxidant compounds, potentially contributing to enhanced tolerance against this atmospheric pollutant. The positive correlation between lignin accumulation and stress severity under O_3_ exposure further substantiates their defensive role [60,61]. Regional variation in lignin response has been documented; for instance, *Fraxinus excelsior* specimens from Tuscany province exhibited significantly elevated lignin content when exposed to O_3_ compared to specimens from Piedmont province [105]. In *Croton floribundus*, O_3_ exposure stimulated the emission of sesquiterpenes, particularly β-caryophyllene, which functions as an effective antioxidant, scavenging O_3_ molecules when plants were subjected to ozone-enriched filtered air versus filtered air alone [143]. Table 1 provides comprehensive information regarding various secondary metabolites and their respective roles as ROS scavengers under oxidative stress conditions.

## 8. Biotechnological Strategies for Enhancing Plant Tolerance to Pollution

**Figure 5:**
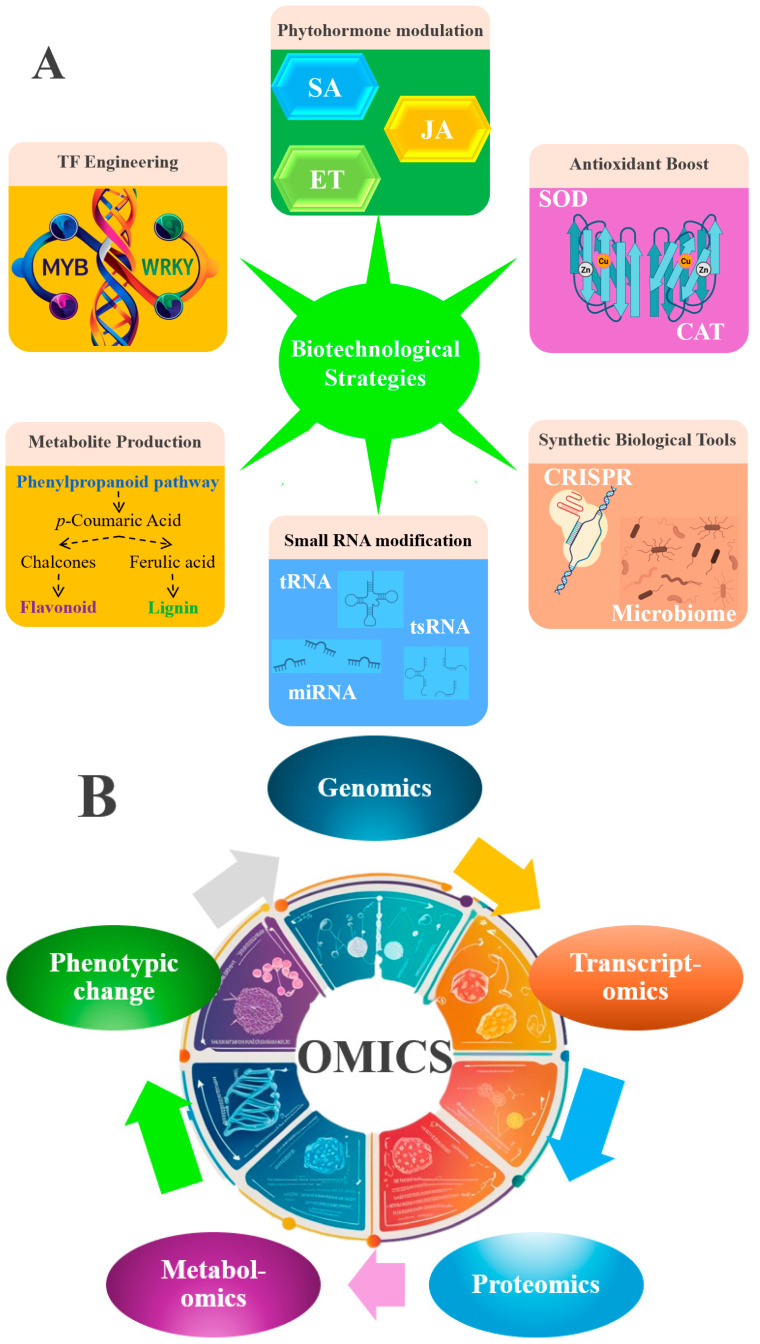
Biotechnological strategies for engineering pollution-tolerant plants. (**A**) Key molecular targets for genetic improvement (Transcription factor (TF) engineering: Overexpression of stress-responsive TFs (MYB, WRKY) to enhance antioxidant pathways. Phytohormone modulation: Genetic manipulation of salicylic acid (SA), jasmonate (JA), and ethylene (ET) signaling. Secondary metabolite production: Upregulation of phenylpropanoid-derived compounds (flavonoids, lignin) via key enzymes (e.g., chalcone synthase). Antioxidant boost: Overexpression of superoxide dismutase (SOD) and catalase (CAT) genes. Synthetic biology tools: CRISPR-based genome editing to integrate multigene traits). (**B**) Omics-guided approaches for stress tolerance breeding: (High-throughput genomics, transcriptomics, proteomics, and metabolomics, miRNAomics, and epigenomics analyses to identify elite cultivars and molecular markers linked to pollution resilience).

Our review demonstrates how biotechnological interventions can systematically enhance plant tolerance to hazardous air pollutants through four synergistic strategies (Figure 5A,B). Overexpression of stress-responsive transcription factors (MYB, WRKY) robustly upregulates lignin biosynthesis pathways, as evidenced in Arabidopsis ozone tolerance studies. Precise modulation of phytohormonal networks (SA/JA/ET) proves equally critical; SA accumulates under ozone exposure by regulating ET and reduces ozone-induced cell death [36]. Metabolic engineering approaches yield particularly promising outcomes, with transgenic upregulation of phenylpropanoid pathway genes increasing protective flavonoid and lignin production, which involves chelating of pollutants [124,144]. When combined with antioxidant system reinforcement (e.g., TaCAT3 wheat lines showing higher heavy metal survival) [68]. Small RNA modifications (e.g., methylation) further expand the biotech toolkit by enabling precision regulation of stress-responsive genes [145]. Emerging single-cell transcriptomics and spatial metabolomics now enable tissue- and subcellular-resolution mapping of these adaptations—revealing compartment-specific mechanisms (e.g., ROS quenching in guard cells or lignin deposition in xylem) that bulk omics miss [146,147]. Complementary omics-driven approaches (Figure 5B) are revolutionizing trait discovery, with multi-omics identifying MYB/WRKY/NAC/bZIP as master regulators of multi-stress responses [148,149,150,151], metabolomics linking specialized compounds like rosmarinic acid to ROS quenching in plants, and proteomics pinpointing RBOHF/APX2 as oxidative stress sentinels. However, a striking translational gap persists—only 15% of omics-predicted biomarkers have been field-validated in polluted environments, underscoring the need for real-world testing of these laboratory-validated solutions. Together, these findings chart a roadmap for developing “green warrior” crops through integrated genetic engineering and precision breeding approaches.

### Multi-Omics Approaches for Decoding Plant Stress Responses

Recent advances in high-throughput “-omics” technologies have revolutionized the study of plant responses to air pollution [152]. These tools provide systemic insights into molecular adaptations: (1) Transcriptomics—RNA sequencing and microarrays identify stress-responsive genes (e.g., WRKY, MYB, RBOHs) under ozone (O_3_) or PM2.5 exposure [1,2], such as Arabidopsis exposed to NO_2_ shows upregulation of ethylene/JA signaling genes (CEJ1, GRX480) [3]. (2) Proteomics—mass spectrometry reveals post-translational modifications (PTMs) and stress-related proteins (e.g., peroxidases, heat shock proteins) [4]. O_3_ stress increases APX2 and glutathione-S-transferase (GST) abundance in Populus [5]. (3) Metabolomics—LC-MS/GC-MS profiles highlight key metabolites (e.g., flavonoids [153,154,155], glutathione, lignin) in pollution-tolerant species [146,147]. PM2.5 induces rosmarinic acid and ascorbate accumulation in *Triticum aestivum* [60,106] (Table 1).

Auxiliary techniques such as 1-epigenomics: DNA methylation (e.g., MET1 gene silencing) modulates stress memory under recurrent O_3_ exposure [156]. 2-miRNAomics: miRNAs (e.g., miR398, miR393) regulate SOD and auxin signaling under heavy metal stress [157]. 3-Microbiome analysis: Rhizosphere microbes enhance pollutant detoxification via symbiotic interactions [158]. While multi-omics data are powerful, their integration remains complex due to technical variability and species-specific responses [152]. Future studies should leverage machine learning to predict cross-talk between omics layers.

## 9. Challenges and Opportunities in Plant-Pollution Interaction Research

1. Combined Stress Responses: Most studies focus on single pollutants, yet plants in urban/industrial areas face simultaneous exposure to multiple stressors (e.g., O_3_ + HMs). Research must elucidate synergistic or antagonistic interactions between pollutants and their integrated effects on plant physiology.
2. Field-to-Lab Translation: While transcriptomic and metabolomic studies reveal stress responses in controlled environments, field validation is scarce. Long-term studies are needed to assess the stability of engineered traits under real-world conditions.
3. Phytohormone Crosstalk: The dynamics of SA, JA, and ethylene signaling under chronic pollution exposure remain poorly understood. Deciphering their spatiotemporal regulation could optimize stress tolerance without compromising growth.
4. Secondary Metabolite Engineering: Despite their protective roles, the metabolic costs of producing flavonoids, lignin, and terpenoids are unclear. Balancing defense and productivity through targeted metabolic engineering is a promising yet underexplored avenue.

## 10. Conclusions

This review comprehensively synthesizes the current understanding of plant defense mechanisms against air pollution, focusing on the pivotal roles of ROS scavenging, antioxidant systems, and molecular signaling pathways. The integration of enzymatic (e.g., SOD, CAT, APX, GR) and non-enzymatic (e.g., flavonoids, glutathione, ascorbate) antioxidant systems is essential for maintaining redox homeostasis and mitigating oxidative damage. Transcription factors such as MYB, WRKY, and NAC, along with MAPK and phytohormone signaling cascades (e.g., salicylic acid, jasmonic acid, ethylene), orchestrate stress-responsive gene networks, enabling plants to adapt to hazardous pollutants. Secondary metabolites, including flavonoids, lignin, and terpenoids, serve as frontline antioxidants and chelators, neutralizing ROS and detoxifying pollutants.

The review also highlights the potential of biotechnological strategies to enhance plant tolerance to pollution, such as the overexpression of ROS-scavenging genes (e.g., *TaCAT3*) and the engineering of phenolic pathways. These approaches, combined with omics-driven insights, offer promising avenues for developing resilient crops capable of thriving in polluted environments. By integrating molecular, metabolic, and biotechnological perspectives, this review provides a roadmap for advancing research and applications in sustainable agriculture under escalating environmental pollution.

## Figures and Tables

**Table 1:** Plant metabolites accumulated in response to air pollutants and their function in stress tolerance and antioxidant defense.

Serial No.	Metabolic Compound	Response to Air Pollution	Heavy Metal Pollution	ROS Quenching	Stress Tolerance Mechanism	Antioxidant Defense	Reference
1	Rosmarinic Acid	Reduces oxidative stress caused by PM and O_3_.	Chelates HMs like cadmium and lead, reducing toxicity.	Direct ROS scavenger enhances antioxidant enzyme.	Strengthen stress tolerance by regulating antioxidant genes.	Activates glutathione-S-transferase (GST) and SOD pathways.	[60,106]
2	Salicylic Acid	Mitigates ozone-induced oxidative stress.	Reduces cadmium and arsenic toxicity by enhancing antioxidant capacity.	Indirect ROS quenching via upregulation of antioxidant enzymes.	Induces systemic acquired resistance (SAR) in plants.	Activates peroxidase (POD) and catalase (CAT) pathways.	[107,108]
3	Syringic Acid	Protects against oxidative damage from air pollutants.	Chelates heavy metals like arsenic stress.	Direct ROS scavenger.	Enhances membrane stability under stress.	Boosts ascorbate peroxidase (APX) activity.	[109,110]
4	Gallic Acid	Reduces oxidative stress from air pollutants.	Chelates heavy metals like chromium and nickel.	Direct ROS scavenger.	Stabilizes cellular membranes and proteins.	Activates SOD and CAT pathways.	[84,111,112]
5	Vanillic Acid	Protect oxidative stress from sulfur dioxide.	Reduces lead and cadmium toxicity.	Indirect ROS quenching via antioxidant enzyme activation.	Enhances lignin biosynthesis for structural defense.	Upregulates POD and APX pathways.	[84,113]
6	Lignin	Acts as a physical barrier against air pollutants.	Binds heavy metals, reducing their bioavailability.	Indirect ROS quenching by reinforcing cell walls.	Provides structural integrity under stress.	Enhances the phenylpropanoid pathway for stress tolerance.	[60,61]
7	p-Coumaric Acid	Reduces oxidative stress from O_3_ and PM.	Chelates heavy metals like cadmium.	Direct ROS scavenger.	Enhances lignin biosynthesis.	Activates PAL (phenylalanine ammonia-lyase) pathway.	[114,115]
8	Caffeic Acid	Protects against oxidative damage from air pollutants.	Chelates heavy metals like iron and copper.	Direct ROS scavenger.	Enhance phenolic compound biosynthesis.	Boosts APX and SOD activity.	[114,116]
9	Ferulic Acid	Reduces oxidative stress from different air pollutants.	Chelates heavy metals like aluminum.	Direct ROS scavenger.	Stabilizes cell walls and membranes.	Activates POD and CAT pathways.	[114,116,117]
10	Isofraxidin	Mitigates oxidative stress from O_3_.	Reduces cadmium toxicity.	Direct ROS scavenger.	Improves lignin biosynthesis.	Activates POD and CAT pathways.	[105]
11	Kaempferol	Reduces oxidative stress from particulate matter.	Chelates heavy metals like lead and cadmium.	Direct ROS scavenger.	Enhances flavonoid biosynthesis.	Activates GST and SOD pathways.	[118]
12	Apigenin	Protects against oxidative damage from air pollutants.	Reduces chromium toxicity.	Direct ROS scavenger.	Strengthens stress tolerance via flavonoid metabolism.	Boosts CAT and APX activity.	[119]
13	Quercetin	Reduces oxidative stress from ozone and nitrogen oxides (NOx).	Chelates heavy metals like cadmium and lead.	Direct ROS scavenger.	Increases flavonoid biosynthesis.	Activates SOD, CAT, and APX pathways.	[118]
14	Quercitrin	Mitigates oxidative damage from air pollutants.	Reduces arsenic toxicity.	Direct ROS scavenger.	Enhance stress tolerance via flavonoid metabolism.	Activates GST and SOD pathways.	[120,121]
15	Rutin	Protects against oxidative stress from sulfur dioxide (SO2).	Chelates heavy metals like cadmium.	Direct ROS scavenger.	Improves flavonoid biosynthesis.	Boosts CAT and APX activity.	[120]
16	Isoquercetrin	Reduces oxidative stress from ozone.	Reduces lead toxicity.	Direct ROS scavenger.	Enhance stress tolerance via flavonoid metabolism.	Activates SOD and CAT pathways.	[105,122]
17	Isoquercetin	Mitigates oxidative damage from air pollutants.	Reduces cadmium toxicity.	Direct ROS scavenger.	Increases flavonoid biosynthesis.	Boosts APX and GST activity.	[105,122]
18	Oleuropein	Protects against oxidative stress from ozone	Chelates heavy metals like copper and zinc.	Direct ROS scavenger.	Enhance phenolic compound biosynthesis.	Activates SOD and CAT pathways.	[105,122]
19	Condensed Tannin	Acts as a physical barrier against air pollutants.	Binds heavy metals, reducing their bioavailability.	Indirect ROS quenching by reinforcing cell walls.	Provides structural integrity under stress.	Enhances the phenylpropanoid pathway for stress tolerance.	[121]
20	Anthocyanins	Reduces oxidative stress from ozone and particulate matter.	Chelates heavy metals like cadmium.	Direct ROS scavenger.	Enhances flavonoid biosynthesis.	Activates SOD, CAT, and APX pathways.	[123,124]
21	Serine	Helps in detoxifying air pollutants by supporting glutathione synthesis.	Reduces heavy metal toxicity by enhancing antioxidant enzyme activity.	Indirect ROS quenching via glutathione production.	Supports cellular metabolism and stress signaling.	Activates glutathione (GSH) biosynthesis pathway.	[62]
22	Threonine	Supports protein synthesis under oxidative stress caused by air pollution.	Chelates heavy metals like cadmium and lead.	Indirect ROS quenching by supporting antioxidant enzyme synthesis.	Boosts protein stability and repair under stress.	Boosts the synthesis of stress-responsive proteins.	[62]
23	Arginine	Reduces oxidative stress from nitrogen oxides (NOx) by producing nitric oxide (NO).	Chelates heavy metals like copper and zinc.	Indirect ROS quenching via nitric oxide (NO) signaling.	Increases stress tolerance through polyamine biosynthesis.	Activates nitric oxide synthase (NOS) and polyamine pathways.	[62]
24	Ornithine	Supports detoxification of air pollutants by participating in the urea cycle.	Reduces heavy metal toxicity by enhancing polyamine biosynthesis.	Indirect ROS quenching via polyamine production.	Enhance cellular repair and stress signaling.	Activates polyamine biosynthesis pathway.	[6]
25	Phenylalanine	Precursor for phenolic compounds that mitigate oxidative stress from air pollution.	Reduces heavy metal toxicity by enhancing lignin and flavonoid biosynthesis.	Indirect ROS quenching via phenolic compound production.	Improves structural defense through lignin biosynthesis.	Activates the phenylpropanoid pathway for antioxidant production.	[7,8]
26	Anthranilate	Supports the synthesis of secondary metabolites that combat oxidative stress.	Reduces HM toxicity by enhancing tryptophan-derived metabolite production.	Indirect ROS quenching via secondary metabolite production.	Enhance stress tolerance through secondary metabolite biosynthesis.	Activates tryptophan metabolism pathway.	[62,105]
27	Histidinol	Precursor for histidine, which plays a role in metal binding and ROS scavenging.	Reduces heavy metal toxicity by chelating metals like nickel and cadmium.	Indirect ROS quenching via histidine production.	Increases metal binding and stress tolerance.	Activates the histidine biosynthesis pathway.	[125]
28	Histidine	Chelates heavy metals and reduces oxidative stress from air pollutants.	Strong metal chelator, reduces the toxicity of nickel, cadmium, and copper.	Direct ROS scavenger and metal chelator.	Enhances metal detoxification and stress tolerance.	Activates metal chelation and antioxidant defense pathways.	[125]
29	Glutamate	Central metabolite in nitrogen metabolism, supports detoxification of air pollutants.	Reduces heavy metal toxicity by enhancing glutathione synthesis.	Indirect ROS quenching via glutathione production.	Boosts nitrogen metabolism and stress signaling.	Activates glutathione (GSH) biosynthesis pathway.	[62,105]
30	Glutamine	Supports the synthesis of antioxidants and stress-responsive proteins.	Reduces heavy metal toxicity by enhancing glutathione synthesis.	Indirect ROS quenching via glutathione production.	Enhances nitrogen metabolism and cellular repair.	Activates glutathione (GSH) biosynthesis and stress-responsive protein pathways.	[62,105]
31	β-Carotene	Protects from oxidative stress caused by O_3_ and PM.	Reduces HM toxicity by scavenging ROS generated by metals like cadmium and lead.	Direct ROS scavenger, protects chlorophyll and membranes.	Protects photosynthetic apparatus and stabilizes membranes.	Activates non-enzymatic antioxidant defense by quenching singlet oxygen and peroxyl radicals.	[13,126,127]
32	α-Pinene	Reduces oxidative stress from O_3_ and nitrogen oxides (NOx).	Reduces HM toxicity by enhancing antioxidant capacity.	Indirect ROS quenching via upregulation of antioxidant enzymes.	Improves membrane stability and reduces lipid peroxidation.	Boosts the activity of SOD and CAT enzymes.	[126]
33	1,8-Cineole	Mitigates oxidative stress from O_3_ and sulfur dioxide (SO_2_).	Decreases HM toxicity by chelating metals like cadmium and lead.	Indirect ROS quenching via antioxidant enzyme activation.	Enhance stress tolerance by stabilizing cellular membranes.	Activates GST and APX pathways.	[127]
34	Camphene	Mitigates oxidative damage from O_3_ and PM.	Reduces heavy metal toxicity by enhancing antioxidant enzyme activity.	Indirect ROS quenching via antioxidant enzyme activation.	Increases membrane stability and reduces oxidative damage.	Boosts SOD and CAT activity.	[126]
35	Quinolizidine	Accumulates in response to ozone (O_3_) to protect against oxidative stress.	Chelating metals like cadmium and nickel.	Indirect ROS quenching via secondary metabolite production.	Improve stress tolerance through alkaloid biosynthesis.	Activates secondary metabolite pathways for stress tolerance.	[122]
36	Piperidine	Mitigates oxidative stress from O_3_ and nitrogen oxides (NOx).	Reduces heavy metal toxicity by chelating metals like lead and cadmium.	Indirect ROS quenching via alkaloid production.	Strengthen stress tolerance through alkaloid biosynthesis.	Activates alkaloid biosynthesis pathways.	[122]
37	Aromadendrene	Protects against oxidative damage from O_3_ and PM.	Lessens HM toxicity by enhancing antioxidant capacity.	Indirect ROS quenching via terpenoid production.	Enhances membrane stability and reduces oxidative damage.	Boosts the activity of antioxidant enzymes like SOD and CAT.	[126]
38	Capsaicin	Reduces oxidative stress from O_3_ and PM.	Scavenging ROS generated by metals like cadmium and Pb.	Direct ROS scavenger.	Boosts stress tolerance through phenolic compound biosynthesis.	Activates the phenylpropanoid pathway for antioxidant production.	[128]
39	Pyridine	Mitigates oxidative stress from O_3_ and nitrogen oxides (NOx).	Chelating metals like nickel and cadmium.	Indirect ROS quenching via alkaloid production.	Improve stress tolerance through alkaloid biosynthesis.	Activates alkaloid biosynthesis pathways.	[122]
40	Pyrimidine	Protects against oxidative damage from O_3_ and PM.	Enhances antioxidant enzyme activity against HM.	Indirect ROS quenching via nucleotide metabolism.	Increase stress tolerance through nucleotide biosynthesis.	Activates nucleotide metabolism pathways for stress tolerance.	[122]

Plant species for each metabolite are documented in the cited references.

## Abbreviations

Symbol	Full Name	Family/Class
MYB	MYELOBLASTOSIS	MYB-domain TFs
WRKY	WRKYGQK domain	WRKY-domain TFs
NAC	NAM/ATAF/CUC (NO APICAL MERISTEM/ARABIDOPSIS TRANSCRIPTION ACTIVATION FACTOR/CUP-SHAPED COTYLEDON)	NAC-domain TFs
bHLH	Basic Helix-Loop-Helix	bHLH-domain TFs
bZIP	Basic Leucine Zipper	bZIP-domain TFs
AP2/ERF	APETALA2/Ethylene-Responsive Factor	AP2/ERF superfamily
ARF	Auxin Response Factor	ARF family
HSF	Heat Shock Factor	HSF family
DREB1/CBF	Dehydration-Responsive Element-Binding Protein 1/C-repeat Binding Factor	AP2/ERF superfamily
ABI3VP1	ABSCISIC ACID INSENSITIVE 3/VIVIPAROUS 1	B3-domain TFs
CCAAT-DR1	CCAAT-box Binding Protein	CCAAT-box TFs
CCAAT-HAP2/3/5	CCAAT-binding Histone-Associated Proteins	CCAAT-box TFs
C2C2-Dof	DNA-binding One Zinc Finger	Dof family
C2C2-CO-like	CONSTANS-like	CO-like family
E2F-DP	E2F-Dimerization Partner	E2F/DP family
C2C2-GATA	GATA-binding	GATA family
C2C2-YABBY	YABBY domain	YABBY family
AtSR	Arabidopsis Stress-Responsive	NF-κB-like TFs
C3H	Cys3His zinc finger	Zinc finger TFs
C2H2	Cys2His2 zinc finger	Zinc finger TFs
EMF1	EMBRYONIC FLOWER 1	Polycomb-group TFs
MADS	MCM1/AGAMOUS/DEFICIENS/SRF	MADS-box TFs
TUB	TUBBY domain	TUBBY family
FIT	FER-LIKE IRON DEFICIENCY-INDUCED TRANSCRIPTION FACTOR	bHLH family
PYE	POPEYE (Iron homeostasis regulator)	bHLH family
HY5	ELONGATED HYPOCOTYL 5	bZIP family
